# Non-RA screening for systemic autoimmune rheumatic diseases using a machine learning model based on physical findings and blood test data

**DOI:** 10.1186/s12911-026-03759-9

**Published:** 2026-08-10

**Authors:** Yosuke Shimada, Satoshi Hori, Yoshiyuki Abe, Minoru Ohno, Masaya Satoh, Mari Sato, Ken Yamaji, Naoto Tamura

**Affiliations:** 1 Infection Control Science, Juntendo University Graduate School of Medicine, Tokyo, Japan; 2 Intelligent Systems Laboratory, SECOM CO., LTD, Tokyo, Japan; 3 Department of Internal Medicine and Rheumatology, Juntendo University School of Medicine, Tokyo, Japan; 4 SECOM Medical System CO., LTD, Tokyo, Japan

**Keywords:** Systemic autoimmune rheumatic diseases, Machine learning, Patient referral

## Abstract

**Background:**

In patients with systemic autoimmune rheumatic diseases (SARDs), particularly patients with non-rheumatoid arthritis (non-RA), the incidence of severe conditions such as interstitial pneumonia and pulmonary arterial hypertension is high. Therefore, early referral of these patients to university hospitals or other institutions capable of comprehensive medical care is desirable. However, the substantial overlap in clinical presentations across SARDs makes differentiation challenging, and objective tools to support disease classification in routine clinical practice remain limited. In this study, a machine learning based classifier was developed to distinguish Rheumatoid Arthritis (RA) from non‑RA conditions using general physical findings and routine blood test data, focusing on patients who were diagnosed with a predefined set of SARDs from a secondary‑care teaching hospital.

**Methods:**

An observational retrospective study was conducted on 571 patients initially diagnosed with SARDs. From the hospital’s clinical information database, patients’ diagnoses, blood test results, and physical findings were extracted from the hospital’s clinical information database to create the dataset. Physical findings were extracted as structured data using natural language processing from medical records written in a free description format by the attending physicians. For patient classification, some non-RA screening model using three classification algorithms were developed: Naive Bayes, Linear Support Vector Machines, and Random Forest.

**Results:**

RA and non-RA diseases share common clinical symptoms, with joint symptoms observed in over 40% of patients with the target diseases. Of the screening models, the random forest model performed the best, achieving a sensitivity of 77.3% and a specificity of 72.5%.

**Conclusions:**

In this single-center retrospective cohort of patients with confirmed SARDs, a machine learning–based classifier using routinely available clinical symptoms and blood test results demonstrated promising internal performance for differentiating RA from non-RA conditions. These findings support the feasibility of machine learning–based classification within this secondary-care setting. Future studies are needed to validate the model in broader clinical populations, including primary care settings, and to evaluate its potential role in supporting referral decision-making.

**Supplementary Information:**

The online version contains supplementary material available at https://doi.org/10.1186/s12911-026-03759-9.

## Background

Systemic Autoimmune Rheumatic Diseases (SARDs), including Rheumatoid Arthritis (RA), Systemic Lupus Erythematosus (SLE), among others, are characterized by systemic and musculoskeletal symptoms associated with autoimmune abnormalities [1]. In Japan, the establishment of a rheumatology specialist system and continuing education through standardized courses ensure the quality of SARDs treatment [2]. For example, 31.8% of RA patients have never visited a medical facility with a rheumatology specialist [3] suggesting that there are serious challenges in referral to specialist care among patients with SARDs. Due to the common clinical features of SARDs, differential diagnosis of individual diseases is challenging [1]. Patients with SARDs other than RA (non-RA diseases) may be at higher risk of severe conditions, including interstitial pneumonia [4] and pulmonary arterial hypertension [5, 6], making early referral to university hospitals capable of multidisciplinary care desirable [7]. One reason for the lack of referrals is the absence of referral criteria for general practitioners. The 2010 American College of Rheumatology/European League Against Rheumatism (ACR/EULAR) criteria for diagnosis of RA require antibody tests such as those for rheumatoid factor and anti-cyclic citrullinated peptide antibody (ACPA) in addition to the presence of joint symptoms [8], which are unfamiliar to non-specialists and may lead to hesitation in conducting these tests. A previous study using machine learning models has proposed methods to predict SARDs based on general physical findings and blood test data [9]. However, while this model could be used to screen for SARDs-like illnesses, it cannot differentiate individual diseases.

Given these challenges, tools that support the differentiation of RA and non‑RA SARDs using routinely available clinical information have the potential to facilitate earlier recognition of patients who may require specialist evaluation. However, the development and evaluation of such tools require reliable diagnostic labels and well characterized clinical data, which are difficult to obtain in primary care settings where diagnostic uncertainty is high. Accordingly, the present study focused on data derived from a secondary care teaching hospital, where definitive diagnoses of SARDs are routinely established. Using general physical findings and routine blood test results obtained at initial presentation, a machine learning based classification model was developed to identify non-RA among suspected SARDs within a predefined group of patients with confirmed SARD diagnoses. While potential applications in primary care remain a future consideration, this study was designed to evaluate internal predictive performance within a controlled secondary care setting.

## Methods

### Study design and population

To develop a machine learning based screening model to Identify non-RA among patients with confirmed SARDs, a retrospective observational study was conducted.

Juntendo University Hospital, one of the largest teaching hospitals in Japan, has a high volume of SARDs patients. In 2018, the annual number of inpatients in the Rheumatology Department, including both newly diagnosed and previously diagnosed patients, was 105 for RA, 140 for Systemic Lupus Erythematosus (SLE), and 59 for Sjögren Syndrome (SjS).

The study included 571 patients who newly confirmed as one of the eight SARDs listed in the differential diagnosis list provided by the Japan College of Rheumatology [10], including RA, SLE, SjS, Systemic Sclerosis (SSc), Polymyalgia Rheumatica (PMR), Dermatomyositis (DM), Mixed Connective Tissue Disease (MCTD), and Anti-neutrophil cytoplasmic antibody Associated Vasculitis (AAV). Among these patients, those with RA were classified as the RA class, while all other diseases were categorized as the non-RA class. Data from the period between May 2008 and December 2019 was collected, and hospitalized patients who had been referred to our department and were newly diagnosed with those eight diseases were included. The dataset was derived from a secondary care clinical setting and therefore does not represent unselected primary care populations.

### Data description

The physical findings and blood test parameters upon patients’ first admission to our department were extracted from the hospital’s clinical information database. Because a wide range of physical findings and laboratory test items were available, the set of candidate predictors was restricted to define the scope of exploration rather than to select variables based on estimated effects. Candidate variables were identified based on rheumatology specialists’ clinical judgment, using the following criteria: (i) clinical relevance to diseases potentially related to the outcome and (ii) feasibility of collection in general clinical practice. No predefined variable list was used, and no data-driven feature selection based on the outcome was performed at this stage. As a result, more than 70 candidate predictors were included in the initial modeling (Table 1). These were used as predictors, and the names of the confirmed diagnoses were used as outcomes to create the dataset.

**Table 1:** Statistical analysis of the clinical characteristics of each class in entire dataset

Category	Variable	All dataset (*n* = 571)	RA (*n* = 153)	Non-RA (*n* = 418)
Demographics	Class (RA/non-RA)	153(26.8%) / 418(73.2%) [571]	--	--
	Disease (RA / SLE / AAV / PMR / MCTD / SjS / DM / SSc)	153(26.8%) / 156(27.3%) / 74(13.0%) / 42(7.4%) / 40(7.0%) / 36(6.3%) / 35(6.1%) / 35(6.1%) [571]	--	--
	Age	53.1 ± 18.1 [100.0%]	59.9 ± 15.8 [100.0%]	50.6 ± 18.3 [100.0%]
	Female sex	468 (82.0%) [100.0%]	115 (75.2%) [100.0%]	353 (84.4%) [100.0%]
General symptoms	Fever	215 [37.7%]	32 [20.9%]	183 [43.8%]
	High-grade fever	8 [1.4%]	1 [0.7%]	7 [1.7%]
	Malaise	91 [15.9%]	17 [11.1%]	74 [17.7%]
	Heat sensation	6 [1.1%]	0 [0]	6 [1.4%]
	Loss of appetite	10 [1.8%]	2 [1.3%]	8 [1.9%]
	Pain (unspecified)	141 [24.7%]	47 [30.7%]	94 [22.5%]
	Syncope	6 [1.1%]	3 [2.0%]	3 [0.7%]
	Gait disturbance	12 [2.1%]	1 [0.7%]	11 [2.6%]
Skin	Alopecia	20 [3.5%]	0 [0]	20 [4.8%]
	Malar rash	5 [0.9%]	1 [0.7%]	4 [1.0%]
	Skin eruption	69 [12.1%]	12 [7.8%]	57 [13.6%]
	Erythema	75 [13.1%]	3 [2.0%]	72 [17.2%]
	Erythema multiforme	34 [6.0%]	2 [1.3%]	32 [7.7%]
	Livedo reticularis	12 [2.1%]	1 [0.7%]	11 [2.6%]
	Photosensitivity	29 [5.1%]	2 [1.3%]	27 [6.5%]
	Purpura	9 [1.6%]	0 [0]	9 [2.2%]
	Palmar erythema	5 [0.9%]	0 [0]	5 [1.2%]
	Scleroderma	28 [4.9%]	2 [1.3%]	26 [6.2%]
	Skin ulcer	11 [1.9%]	2 [1.3%]	9 [2.2%]
	Pachyonychia	7 [1.2%]	1 [0.7%]	6 [1.4%]
Respiratory	Cough	48 [8.4%]	10 [6.5%]	38 [9.1%]
	Dyspnea	34 [6.0%]	7 [4.6%]	27 [6.5%]
	Exertional dyspnea	21 [3.7%]	5 [3.3%]	16 [3.8%]
	Pleural effusion	10 [1.8%]	1 [0.7%]	9 [2.2%]
	Chest pain	25 [4.4%]	6 [3.9%]	19 [4.5%]
	Chest discomfort	6 [1.1%]	2 [1.3%]	4 [1.0%]
Edema	Edema (generalized)	50 [8.8%]	7 [4.6%]	43 [10.3%]
	Peripheral edema	49 [8.6%]	6 [3.9%]	43 [10.3%]
	Facial edema	7 [1.2%]	0 [0]	7 [1.7%]
	Eyelid edema	8 [1.4%]	0 [0]	8 [1.9%]
Neurologic	Altered consciousness	6 [1.1%]	0 [0]	6 [1.4%]
	Seizure	5 [0.9%]	0 [0]	5 [1.2%]
	Headache	44 [7.7%]	3 [2.0%]	41 [9.8%]
	Ocular pain	5 [0.9%]	0 [0]	5 [1.2%]
	Hearing impairment	7 [1.2%]	0 [0]	7 [1.7%]
	Hypesthesia	42 [7.4%]	3 [2.0%]	39 [9.3%]
Musculoskeletal	Myalgia	62 [10.9%]	5 [3.3%]	57 [13.6%]
	Joint symptom	309 [54.1%]	111 [72.5%]	198 [47.4%]
	Polyarthritis	13 [2.3%]	4 [2.6%]	9 [2.2%]
Gastrointestinal	Abdominal pain	21 [3.7%]	3 [2.0%]	18 [4.3%]
	Diarrhea	33 [5.8%]	7 [4.6%]	26 [6.2%]
	Dysphagia	6 [1.1%]	0 [0]	6 [1.4%]
	Oral symptoms	46 [8.1%]	9 [5.9%]	37 [8.9%]
	Epistaxis	7 [1.2%]	1 [0.7%]	6 [1.4%]
Other symptoms	Raynaud phenomenon	24 [4.2%]	1 [0.7%]	23 [5.5%]
	Vulvovaginitis	5 [0.9%]	2 [1.3%]	3 [0.7%]
	Neck pain	10 [1.8%]	1 [0.7%]	9 [2.2%]
	Alveolar osteitis	10 [1.8%]	0 [0]	10 [2.4%]
Hematology	White blood cell count	7.4 ± 4.5 [99.8%]	7.2 ± 3.4 [100.0%]	7.4 ± 4.8 [99.8%]
	Eosinophil count	258.4 ± 2242.2 [99.6%]	168.3 ± 674.7 [99.3%]	291.3 ± 2587.5 [99.8%]
	Platelet count	267.8 ± 120.8 [99.6%]	284.9 ± 126.7 [99.3%]	261.6 ± 118.1 [99.8%]
	Reticulated platelet count	2.6 ± 2.7 [81.6%]	2.2 ± 2.3 [68.0%]	2.7 ± 2.8 [86.6%]
	PDW	11.1 ± 1.8 [95.8%]	10.8 ± 1.5 [97.4%]	11.2 ± 1.9 [95.2%]
	MPV	9.9 ± 0.9 [95.8%]	9.7 ± 0.8 [97.4%]	9.9 ± 1.0 [95.2%]
	PCT	0.3 ± 0.1 [95.8%]	0.3 ± 0.1 [97.4%]	0.3 ± 0.1 [95.2%]
	MCV	88.7 ± 6.9 [99.8%]	91.1 ± 6.7 [100.0%]	87.9 ± 6.8 [99.8%]
	MCH	29.2 ± 2.8 [99.8%]	30.0 ± 2.9 [100.0%]	28.9 ± 2.7 [99.8%]
Inflammation	C-reactive protein	3.4 ± 5.6 [99.6%]	3.4 ± 5.0 [99.3%]	3.3 ± 5.8 [99.8%]
	ESR	56.4 ± 40.7 [93.2%]	49.8 ± 41.4 [96.1%]	58.9 ± 40.2 [92.1%]
	Fibrinogen	442.3 ± 172.1 [60.1%]	448.0 ± 167.5 [43.8%]	440.9 ± 173.5 [66.0%]
Biochemistry	Total protein	6.9 ± 0.9 [99.6%]	7.0 ± 0.7 [99.3%]	6.9 ± 0.9 [99.8%]
	LDH	253.2 ± 169.9 [99.6%]	226.2 ± 102.9 [99.3%]	263.0 ± 187.7 [99.8%]
	Serum calcium	8.9 ± 0.6 [86.7%]	9.0 ± 0.5 [77.8%]	8.9 ± 0.7 [90.0%]
	Serum phosphate	3.5 ± 0.9 [47.5%]	3.4 ± 0.7 [33.3%]	3.5 ± 0.9 [52.6%]
	Serum creatinine	0.7 ± 0.6 [99.6%]	0.7 ± 0.4 [99.3%]	0.8 ± 0.7 [99.8%]
	BUN	16.7 ± 11.2 [99.6%]	16.4 ± 7.1 [99.3%]	16.8 ± 12.3 [99.8%]
	eGFR	92.0 ± 37.7 [99.6%]	93.3 ± 34.1 [99.3%]	91.5 ± 39.0 [99.8%]
	Serum uric acid	4.8 ± 1.7 [98.9%]	4.5 ± 1.6 [98.0%]	4.9 ± 1.7 [99.3%]
	Serum chloride	103.2 ± 3.6 [99.6%]	103.3 ± 3.2 [99.3%]	103.2 ± 3.7 [99.8%]
Lipids	Triglycerides	125.9 ± 79.1 [98.9%]	102.1 ± 52.5 [98.7%]	134.5 ± 85.2 [99.0%]
	LDL/HDL ratio	2.3 ± 1.1 [81.8%]	2.0 ± 1.0 [73.9%]	2.4 ± 1.1 [84.7%]
	Hemoglobin A1c	5.9 ± 0.9 [71.5%]	6.0 ± 1.0 [63.4%]	5.9 ± 0.8 [74.4%]
Urinalysis	Urine protein	50.8 ± 140.0 [76.5%]	21.9 ± 15.2 [54.9%]	57.7 ± 154.8 [84.4%]
Coagulation	Prothrombin Time	14.5 ± 2.4 [91.9%]	14.6 ± 1.5 [79.7%]	14.5 ± 2.6 [96.4%]
Immunology	Immunoglobulin G	1581.4 ± 665.5 [99.5%]	1449.1 ± 436.7 [98.7%]	1629.2 ± 725.4 [99.8%]

Continuous variables are expressed as means and standard deviations. Categorical variables are expressed as number. The observed proportion (%) for each group is indicated in square brackets []

The clinical course and symptom progression until the first admission were recorded by the attending physicians in a free description format. Since they were documented prior to hospitalization, it is ensured that these data represent information obtained before the confirmed diagnosis. The positive physical findings were extracted from these descriptions as structured data using the Adverse Effect eXtractor [11].

### Summary of patient characteristics

To understand the distribution of predictor variables, the statistical measures of clinical characteristics were calculated for each class (RA vs. non-RA). For binary variables, the count and percentage were calculated, while for continuous variables, the mean and variance were computed. To support the interpretation of disease characteristics, statistical measures of clinical characteristics were calculated for each of the eight diseases. Chi-square tests were performed, and only variables showing statistically significant differences at a significance level of 5% were extracted for descriptive purposes. All analyses were performed using the entire dataset.

### Model development and comparison

Binary classification models to discriminate RA from non‑RA conditions were constructed. Several machine learning algorithms were compared to evaluate their predictive performance. The primary methods included (1) Naïve Bayes (NB), (2) Linear Support Vector Machine (Linear SVM), and (3) Random Forest (RF) classifiers. For the random forest models, we constructed several variants to examine the impact of different preprocessing strategies. These variants included: (3a) a model using all available predictors (RF-all); (3b) a model excluding physical examination findings (RF-noPhys); (3c) a model excluding blood test results (RF-noLab); (3d) a model in which missing physical examination findings were imputed with the most frequent value and encoded as binary positive/negative variables (RF-binPhys), whereas in the other models physical examination findings were represented as categorical variables with three levels (positive, negative, and not recorded); and (3e) a two‑stage model in which an eight‑class Random Forest classifier was used in the first stage and a logistic regression classifier in the second stage to predict RA versus non‑RA from the first‑stage class‑probability outputs (RF‑stacked). All performance metrics were calculated at the patient level for binary classification of non‑RA versus RA, with non-RA defined as the positive class. True positives, false positives, true negatives, and false negatives were defined based on the final binary predictions. In the RF‑stacked model, the multiclass classifier was used solely to generate class‑probability features, and all non‑RA diagnostic categories were grouped together for evaluation.

These models were subsequently evaluated using the nested cross-validation framework described below.

### Feature engineering

Because the recording of physical examination findings and blood tests depended on the attending physician, missingness occurred in a non-systematic manner. Physical examination findings were handled as described above. For blood tests, preprocessing differed by algorithm: naïve Bayes was applied without imputation because it can accommodate missing values, whereas SVM and random forest required complete data and therefore received median imputation for continuous variables and mode imputation for binary variables.

For the naïve Bayes model, conditional independence among predictors was explicitly assumed. To support this assumption, highly correlated pairs of variables were identified, and one variable from each pair was removed. The correlation threshold used for this filtering was included as a tunable hyperparameter within the grid search, and when multiple correlated variables met the threshold, the variable appearing later in the data table was excluded.

### Nested cross validation

In this study, nested cross-validation was employed to obtain an unbiased estimate of the generalization performance of the models. First, an outer resampling procedure was performed on the entire dataset, dividing it into multiple outer folds. For each outer fold, one-fold was designated as the outer test set, while the remaining folds were used as the outer training set. This outer resampling framework provides a robust basis for mitigating bias in the final performance evaluation.

Subsequently, inner resampling was applied within each outer training set to optimize the hyperparameters. In this inner procedure, the outer training set was further partitioned into several inner folds, which were used for model selection. Specifically, in each inner fold, candidate models were trained on the inner training set and evaluated on the inner validation set. This inner loop enabled the identification of the optimal hyperparameter configuration. A grid search over a predefined lattice of candidate values was used for the hyperparameter search, and the ROC AUC, which is relatively robust to class imbalance and independent of decision thresholds, was adopted as the performance metric for evaluating each candidate setting. For the RF‑stacked model, the hyperparameter optimization was applied only to the first‑stage RF classifier using ROC AUC as the objective metric. The second‑stage logistic regression classifier was fitted without additional hyperparameter tuning. In addition, the decision threshold was determined by maximizing the Youden Index (sensitivity + specificity − 1) on the inner validation set, with the search constrained to thresholds yielding sensitivity between 0.7 and 0.9, thereby achieving a balanced trade-off between sensitivity and specificity. This constraint was introduced to ensure clinically acceptable sensitivity for a screening-oriented model.

Using the hyperparameters selected in the inner loop, a model was retrained on the entire outer training set and subsequently evaluated on the outer test set to obtain an unbiased estimate of its generalization performance. This procedure was repeated across all outer folds to ensure stable and reliable estimation of model performance on unseen data. During evaluation on the outer test set, the decision threshold determined in the inner loop was applied. At this stage, several performance metrics were calculated to comprehensively evaluate predictive performance, including sensitivity, specificity, positive predictive value (PPV), negative predictive value (NPV), accuracy, and the area under the ROC curve (ROC AUC).

### Handling imbalanced data

In this dataset, the distribution between the two classes was imbalanced, with non-RA classes having substantially more samples than RA class. Using Random Forest on such imbalanced data tends to result in overfitting, with the model biased towards diseases with more cases. To mitigate this, we utilized case weights provided by the machine learning library to compensate for the minority class [12].

### Aggregation and interpretation of model outputs

Nested cross-validation was used to generate ten models and their corresponding performance metrics for each method, derived from the ten outer folds. For each metric, the mean and the 95% confidence interval were computed, assuming a t‑distribution of the fold‑level estimates. To aggregate the hyperparameters obtained across the ten outer folds, the mode was extracted for each hyperparameter as the representative value. In addition, feature importance was assessed using permutation importance to quantify the contribution of each predictor to the model output. Because permutation importance is a relative measure, importance ranks were used for evaluation. As ranks varied across the 10 outer folds, feature importance was summarized using the mean and standard deviation of the ranks.

To assess model calibration, predictions obtained from the outer test sets were aggregated across all outer folds. These out-of-fold (OOF) predictions represent model outputs for samples that were not used during training, thereby providing an unbiased basis for evaluation. Using the aggregated OOF predictions, calibration performance was assessed by estimating the calibration intercept and calibration slope, constructing calibration plots, and calculating the Brier score. In addition, decision curve analysis was performed across a range of threshold probabilities to evaluate whether the model provides greater clinical benefit than treating all or no patients.

## Results

Table 1 shows the aggregated clinical characteristics and their comparisons between the RA and non‑RA groups in the whole dataset. Joint symptoms were common physical findings across all diseases, observed in more than 30% of patients, even in patients with less frequent diseases. Similarly, fever was observed in more than 18% of patients.

Among the seven evaluated models, the RF-all model achieved the highest overall discriminative performance, with an ROC AUC of 84.7%, along with an accuracy of 76.0%, sensitivity of 77.3%, specificity of 72.5%, PPV of 88.5%, and NPV of 55.9 (Table 2). In the comparison across modelling approaches, both the NB and Linear SVM models showed performance broadly comparable to that of the RF-all model in terms of sensitivity and specificity, although the RF-all model demonstrated modest improvements in overall discrimination as reflected by a higher ROC AUC. Among the RF variant models, the performance of the RF-noPhys model showed the most notable decline compared with the RF-all model. In particular, the omission of physical examination variables resulted in a reduction in overall discriminative performance, with a decrease of approximately 5% in ROC AUC. With respect to the RF-all model, the most frequently selected hyperparameter values across the 10 outer folds were as follows: the number of predictors sampled at each split (mtry) was 21, the number of decision trees (trees) was 1,000, and the minimum node size (min_n) was 11. The hyperparameters for the other models, as well as the candidate values evaluated during grid tuning, are shown in Table 3. To examine the variables that contributed most strongly to the RF-all model, the top 15 variables were summarized based on the mean and standard deviation of their importance ranks across the 10 outer folds, as shown in Table 4. Among these variables, joint symptoms erythema, and fever consistently ranked highly on average, with relatively small standard deviations, indicating stable importance across folds.

**Table 2:** The performance of each model

Model name	ROC AUC	Accuracy	Sensitivity	Specificity	NPV	PPV	Brier score
(1) NB	79.1 (74.3 ~ 83.9)	74.3 (68.6 ~ 79.9)	74.7 (66.9 ~ 82.4)	73.2 (67.8 ~ 78.7)	53.4 (45.6 ~ 61.2)	88.3 (86.0 ~ 90.7)	18.6 (15.4 ~ 21.9)
(2) Linear SVM	83.0 (79.5 ~ 86.6)	74.8 (71.2 ~ 78.4)	75.4 (69.4 ~ 81.4)	73.1 (62.0 ~ 84.3)	52.8 (48.2 ~ 57.4)	89.0 (85.3 ~ 92.7)	14.4 (13.3 ~ 15.4)
(3a) RF-all	84.7 (80.0 ~ 89.4)	76.0 (70.0 ~ 82.0)	77.3 (70.1 ~ 84.5)	72.5 (63.4 ~ 81.5)	55.9 (46.2 ~ 65.6)	88.5 (84.9 ~ 92.1)	14.8 (13.4 ~ 16.2)
(3b) RF-noPhys	80.1 (75.2 ~ 85.0)	72.7 (67.5 ~ 77.8)	73.2 (67.5 ~ 78.9)	71.0 (61.3 ~ 80.8)	49.8 (43.2 ~ 56.3)	87.5 (83.7 ~ 91.3)	17.8 (17.0 ~ 18.6)
(3c) RF-noLab	82.8 (78.7 ~ 87.0)	73.6 (67.6 ~ 79.5)	73.0 (64.0 ~ 81.9)	75.1 (66.6 ~ 83.5)	52.9 (43.9 ~ 61.8)	89.1 (86.5 ~ 91.8)	16.5 (14.2 ~ 18.7)
(3d) RF-binPhys	83.2 (78.4 ~ 88.0)	76.9 (72.4 ~ 81.4)	77.8 (72.9 ~ 82.6)	74.5 (66.6 ~ 82.4)	55.8 (49.2 ~ 62.4)	89.4 (86.1 ~ 92.6)	17.7 (15.5 ~ 19.9)
(3e) RF-stacked	85.0 (79.6 ~ 90.3)	80.0 (75.4 ~ 84.7)	89.0 (83.2 ~ 94.9)	55.7 (41.0 ~ 70.3)	69.3 (54.8 ~ 83.7)	85.0 (80.8 ~ 89.2)	14.4 (13.0 ~ 15.7)

All values are reported as “mean (95% confidence interval)”

**Table 3:** Selected hyperparameters and candidate values on grid

Model Name	Hyperparameter	Selected Value	Candidate values on grid
NB	smoothness	1.5	0.5 / 0.75 / 1 / 1.25 / 1.5
	Laplace	0	0 / 0.75 / 1.5 / 2.25 / 3
	threshold	0.5	0 / 0.25 / 0.5 / 0.75 / 1
Linear SVM	cost	0.177	0.000977 / 0.0131 / 0.177 / 2.38 / 32
	margin	0	0 / 0.05 / 0.1 / 0.15 / 0.2
RF-all	mtry	21	1 / 21 / 42 / 62 / 83
	trees	1000	1 / 500 / 1000 / 1500 / 2000
	min_n	11	2 / 11 / 21 / 30 / 40
RF-noPhys	mtry	1	1 / 21 / 42 / 62 / 83
	trees	1000	1 / 500 / 1000 / 1500 / 2000
	min_n	2	2 / 11 / 21 / 30 / 40
RF-noLab	mtry	21	1 / 21 / 42 / 62 / 83
	trees	2000	1 / 500 / 1000 / 1500 / 2000
	min_n	40	2 / 11 / 21 / 30 / 40
RF-binPhys	mtry	1	1 / 21 / 42 / 62 / 83
	trees	1000	1 / 500 / 1000 / 1500 / 2000
	min_n	2	2 / 11 / 21 / 30 / 40
RF-stacked	mtry	21	1 / 21 / 42 / 62 / 83
	trees	1000	1 / 500 / 1000 / 1500 / 2000
	min_n	11	2 / 11 / 21 / 30 / 40

For the Random Forest model, the following hyperparameters were tuned: mtry (the number of predictors considered at each split), trees (the number of decision trees), and min_n (the minimum number of samples required to split an internal node). For the linear Support Vector Machine model, the tuned hyperparameters included cost (the penalty parameter for misclassification) and margin (the distance from the decision boundary). For the Naïve Bayes model, the tuned hyperparameters were smoothness (the smoothing parameter for probability density estimation), Laplace (the Laplace correction for zero-frequency issues), and threshold (the correlation coefficient threshold used for variable exclusion)

**Table 4:** Top 15 feature importances in RF-all model

Variable	Mean of rank	Standard deviation of rank
Joint symptom	1.8	1.75
Age	4.2	3.49
Fever	9.3	5.46
Erythema	18.4	38.90
Eosinophil count	19.8	39.90
MCH	21.4	36.50
MCV	23.1	38.50
Immunoglobulin G	24.8	35.10
LDH	25.0	37.60
Hypesthesia	26.1	30.60
LDL/HDL ratio	28.3	50.80
Myalgia	28.9	36.90
Scleroderma	33.6	24.90
Prothrombin Time	35.4	50.20
Urine protein	35.8	47.20

In a supplementary analysis, the distribution of physical findings was examined across the eight diseases. Several physical findings showed statistically significant differences among disease groups based on chi-square tests (Table 5). Among these findings, for example, erythema, hypesthesia, and peripheral edema were observed less frequently in RA, whereas they were more common in non-RA diseases.

**Table 5:** Distribution of physical findings by disease (significant findings only)

Category	Variable	SLE	RA	AAV	PMR	MCTD	SjS	DM	SSc	*P* Value
General symptoms	Fever	89 (58.6%) [152]	32 (22.9%) [140]	37 (52.9%) [70]	11 (28.2%) [39]	20 (50.0%) [40]	12 (41.4%) [29]	6 (17.6%) [34]	8 (23.5%) [34]	< 0.001
	Pain (unspecified)	28 (18.4%) [152]	47 (33.6%) [140]	22 (31.4%) [70]	22 (56.4%) [39]	4 (10.0%) [40]	6 (20.7%) [29]	8 (23.5%) [34]	4 (11.8%) [34]	< 0.001
Skin	Alopecia	18 (11.8%) [152]	0 (0.0%) [140]	0 (0.0%) [70]	0 (0.0%) [39]	1 (2.5%) [40]	0 (0.0%) [29]	1 (2.9%) [34]	0 (0.0%) [34]	< 0.001
	Skin eruption	28 (18.4%) [152]	12 (8.6%) [140]	5 (7.1%) [70]	0 (0.0%) [39]	4 (10.0%) [40]	1 (3.4%) [29]	17 (50.0%) [34]	2 (5.9%) [34]	< 0.001
	Erythema	39 (25.7%) [152]	3 (2.1%) [140]	9 (12.9%) [70]	1 (2.6%) [39]	2 (5.0%) [40]	3 (10.3%) [29]	16 (47.1%) [34]	2 (5.9%) [34]	< 0.001
	Erythema multiforme	30 (19.7%) [152]	2 (1.4%) [140]	0 (0.0%) [70]	0 (0.0%) [39]	1 (2.5%) [40]	0 (0.0%) [29]	1 (2.9%) [34]	0 (0.0%) [34]	< 0.001
	Livedo reticularis	2 (1.3%) [152]	1 (0.7%) [140]	3 (4.3%) [70]	0 (0.0%) [39]	0 (0.0%) [40]	1 (3.4%) [29]	4 (11.8%) [34]	1 (2.9%) [34]	0.006
	Photosensitivity	24 (15.8%) [152]	2 (1.4%) [140]	1 (1.4%) [70]	0 (0.0%) [39]	2 (5.0%) [40]	0 (0.0%) [29]	0 (0.0%) [34]	0 (0.0%) [34]	< 0.001
	Scleroderma	3 (2.0%) [152]	2 (1.4%) [140]	0 (0.0%) [70]	0 (0.0%) [39]	5 (12.5%) [40]	0 (0.0%) [29]	1 (2.9%) [34]	17 (50.0%) [34]	< 0.001
Respiratory	Cough	8 (5.3%) [152]	10 (7.1%) [140]	10 (14.3%) [70]	0 (0.0%) [39]	5 (12.5%) [40]	4 (13.8%) [29]	7 (20.6%) [34]	4 (11.8%) [34]	0.018
	Exertional dyspnea	1 (0.7%) [152]	5 (3.6%) [140]	2 (2.9%) [70]	0 (0.0%) [39]	5 (12.5%) [40]	1 (3.4%) [29]	4 (11.8%) [34]	3 (8.8%) [34]	0.003
Edema	Peripheral edema	14 (9.2%) [152]	6 (4.3%) [140]	7 (10.0%) [70]	1 (2.6%) [39]	8 (20.0%) [40]	4 (13.8%) [29]	4 (11.8%) [34]	5 (14.7%) [34]	0.049
	Eyelid edema	4 (2.6%) [152]	0 (0.0%) [140]	1 (1.4%) [70]	0 (0.0%) [39]	0 (0.0%) [40]	0 (0.0%) [29]	3 (8.8%) [34]	0 (0.0%) [34]	0.011
Neurologic	Headache	18 (11.8%) [152]	3 (2.1%) [140]	10 (14.3%) [70]	6 (15.4%) [39]	2 (5.0%) [40]	3 (10.3%) [29]	2 (5.9%) [34]	0 (0.0%) [34]	0.006
	Hearing impairment	1 (0.7%) [152]	0 (0.0%) [140]	5 (7.1%) [70]	0 (0.0%) [39]	0 (0.0%) [40]	0 (0.0%) [29]	0 (0.0%) [34]	1 (2.9%) [34]	0.001
	Hypesthesia	10 (6.6%) [152]	3 (2.1%) [140]	14 (20.0%) [70]	2 (5.1%) [39]	5 (12.5%) [40]	5 (17.2%) [29]	1 (2.9%) [34]	2 (5.9%) [34]	< 0.001
Musculoskeletal	Myalgia	16 (10.5%) [152]	5 (3.6%) [140]	8 (11.4%) [70]	11 (28.2%) [39]	8 (20.0%) [40]	1 (3.4%) [29]	13 (38.2%) [34]	0 (0.0%) [34]	< 0.001
	Joint symptom	98 (64.5%) [152]	111 (79.3%) [140]	24 (34.3%) [70]	22 (56.4%) [39]	19 (47.5%) [40]	11 (37.9%) [29]	12 (35.3%) [34]	12 (35.3%) [34]	< 0.001
Gastrointestinal	Dysphagia	0 (0.0%) [152]	0 (0.0%) [140]	1 (1.4%) [70]	0 (0.0%) [39]	1 (2.5%) [40]	1 (3.4%) [29]	3 (8.8%) [34]	0 (0.0%) [34]	< 0.001
	Oral symptoms	31 (20.4%) [152]	9 (6.4%) [140]	1 (1.4%) [70]	1 (2.6%) [39]	1 (2.5%) [40]	1 (3.4%) [29]	1 (2.9%) [34]	1 (2.9%) [34]	< 0.001
Other symptoms	Raynaud phenomenon	6 (3.9%) [152]	1 (0.7%) [140]	1 (1.4%) [70]	0 (0.0%) [39]	10 (25.0%) [40]	2 (6.9%) [29]	2 (5.9%) [34]	2 (5.9%) [34]	< 0.001
	Neck pain	0 (0.0%) [152]	1 (0.7%) [140]	3 (4.3%) [70]	5 (12.8%) [39]	0 (0.0%) [40]	0 (0.0%) [29]	1 (2.9%) [34]	0 (0.0%) [34]	< 0.001
	Alveolar osteitis	1 (0.7%) [152]	0 (0.0%) [140]	0 (0.0%) [70]	0 (0.0%) [39]	1 (2.5%) [40]	6 (20.7%) [29]	1 (2.9%) [34]	1 (2.9%) [34]	< 0.001

The confusion matrix for the first-stage eight-class classification in the RF-stacked model is shown in Table 6. SLE and RA tended to be classified correctly. In contrast, classification performance for MCTD and SjS was poorer, with many cases being misclassified as SLE. In addition, Table 7 presents the false-negative rates of the RF-all model stratified by disease subtype. The false-negative rates, defined as the proportion of non‑RA cases misclassified as RA, varied across diseases, with particularly high rates observed for PMR and SjS.

**Table 6:** Confusion matrix for eight-class classification in the RF-stacked model

		Predicted label							
		SLE	RA	AAV	PMR	MCTD	SjS	DM	SSc
Truth label	SLE	134	19	2	0	1	0	0	0
	RA	16	124	7	5	0	0	1	0
	AAV	12	33	27	1	0	0	0	1
	PMR	3	29	7	3	0	0	0	0
	MCTD	27	10	0	0	0	0	1	2
	SjS	14	18	4	0	0	0	0	0
	DM	15	10	2	0	1	0	6	1
	SSc	3	18	6	0	0	0	0	8

**Table 7:** False positive rate each disease in the RF-all model, with non-RA defined as the positive class

True label	Predicted as non-RA	Predicted as RA	False Positive Rate
SLE	153	3	1.92
RA	61	92	39.87
AAV	64	10	13.51
PMR	26	16	38.10
MCTD	38	2	5.00
SjS	24	12	33.33
DM	33	2	5.71
SSc	28	7	20.00

The results of predictor preprocessing are summarized in Tables 8 and 9. Because preprocessing was performed independently within each outer fold, the imputed values and the set of retained predictors varied depending on the data included in each fold. Table 8 presents the imputed values for missing data in fold 1 of the RF-all model as a representative example. Table 9 summarizes the results of correlation-based feature selection for the naïve Bayes model. As the correlation threshold was determined through fine tuning, the list of predictors excluded in fold 1 is shown for the model with a threshold of 0.5, which was the most frequently selected value across tuning results.

**Table 8:** List of imputed values for the Linear SVM and RF models (results from a selected fold)

Variables	Imputed Value
Age	53.9
White blood cell count	6.60
Eosinophil count	4.06
Platelet count	252
Reticulated platelet count	1.90
PDW	10.9
MPV	9.80
PCT	0.25
MCV	89.1
MCH	29.5
C-reactive protein	0.60
ESR	46.0
Fibrinogen	402
Total protein	7.00
LDH	209
Serum calcium	9.00
Serum phosphate	3.50
Serum creatinine	0.570
BUN	14.0
eGFR	90.0
Serum uric acid	4.60
Serum chloride	104
Triglycerides	107
LDL/HDL ratio	2.10
Hemoglobin A1c	5.80
Urine protein	3.00
Prothrombin Time	14.1
Immunoglobulin G	1456

**Table 9:** Variables excluded due to correlation in the NB model

Variables
Urine protein
PDW
MPV
MCH
NA
ESR
C-reactive protein
Platelet count
Eosinophil count
Prothrombin Time
Serum creatinine
eGFR
Serum uric acid
Total protein

## Discussion

In this study, a machine learning based screening model was developed to differentiate patients with non-RA conditions among individuals referred for suspected SARDs. In this clinical context, non‑RA conditions have been reported to be associated with a higher risk of severe complications [7], underscoring the importance of their accurate identification. Using a retrospective, single center dataset, the model achieved a sensitivity of 77.3% and a specificity of 72.5% in internal validation. Because non‑RA was defined as the positive class, sensitivity corresponds to the detection rate of non‑RA conditions. These results indicate that routinely collected physical findings and blood test data contain informative patterns that can support discrimination between RA and non‑RA conditions within this defined secondary care population.

It is known that RA and non-RA diseases share common clinical symptoms [1], and in this study, joint symptoms and fever were frequently observed across two classes (Table 1). Therefore, it is challenging to differentiate diseases based on a single symptom, highlighting the potential need for improved screening approaches in earlier clinical settings. The aim of this study was to explore whether non-RA diseases could be identified based on physical findings and blood test results within this study population.

The RF-all model achieved an overall ROC AUC of 84.7%, with a sensitivity of 77.3% and specificity of 72.5%, indicating good discriminative performance under the optimized decision threshold. The positive predictive value was high (89.0%), suggesting that patients classified as positive are likely to have non-RA SARDs. In contrast, the negative predictive value was relatively low (55.9%), indicating limited ability to reliably exclude disease.

**Fig. 1:**
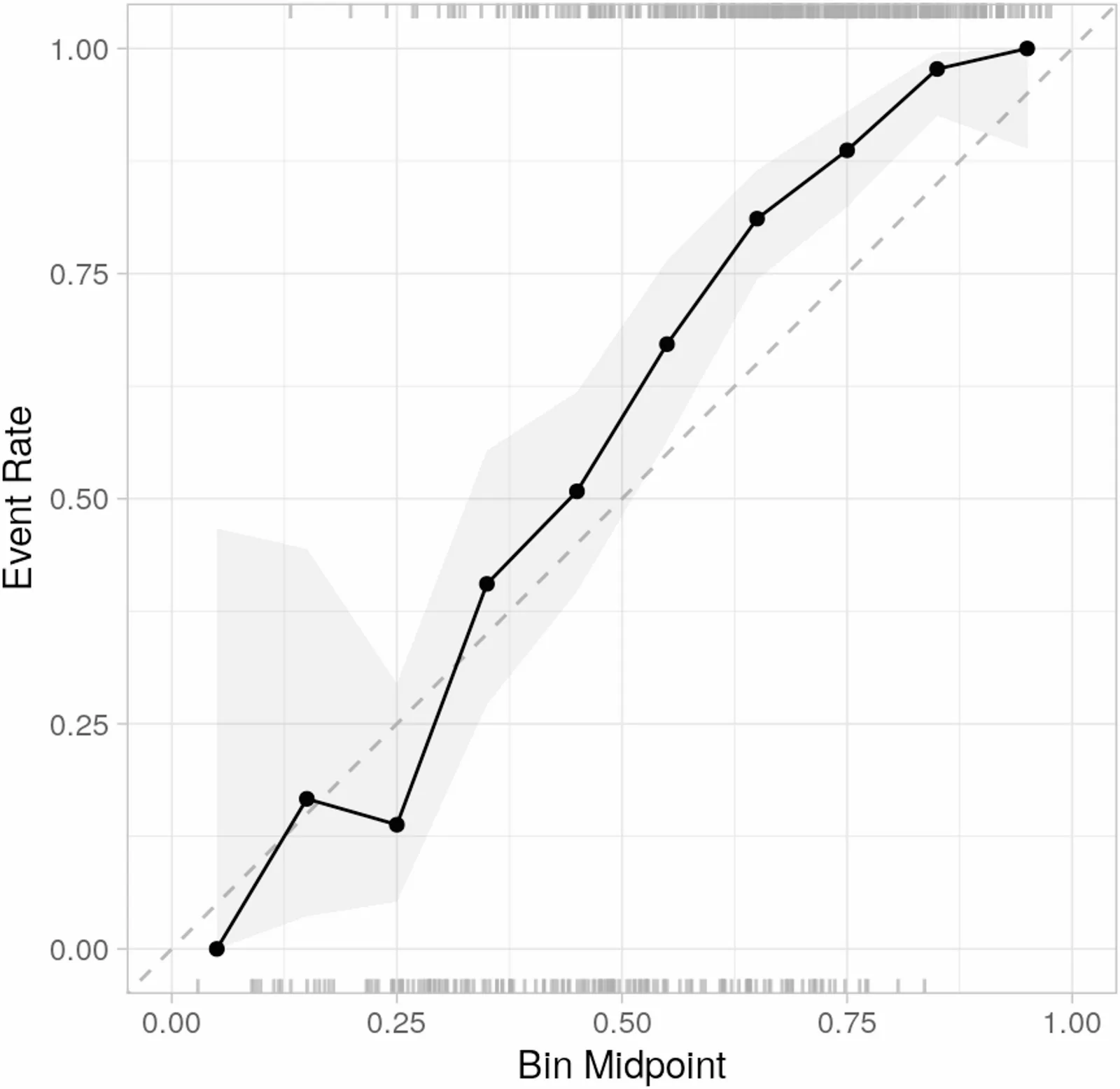
Calibration plot for RF-all model Calibration plot of the model based on out-of-fold predictions. The x-axis represents the predicted probability, and the y-axis shows the observed outcome frequency within each bin. Predictions were grouped into 10 bins, and points indicate the agreement between predicted and observed probabilities. The diagonal line represents perfect calibration, with closer alignment indicating better calibration performance. The gray shaded area represents the uncertainty around the estimated observed probabilities (confidence intervals). The vertical bars indicate the distribution of predicted probabilities within each bin

**Fig. 2:**
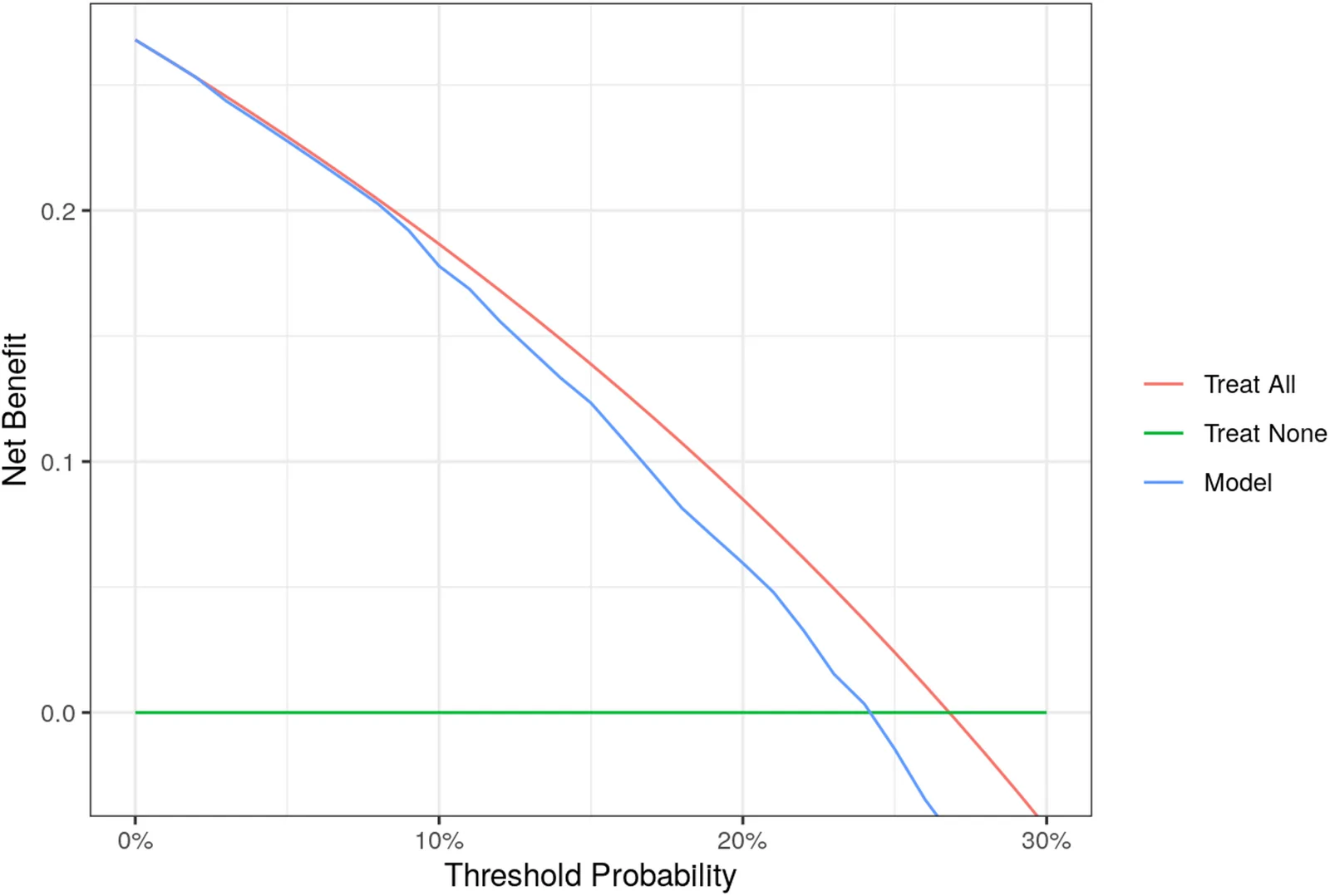
Decision-curve analysis for RF-all model. Decision curve analysis of the model across threshold probabilities from 0 to 0.3. The x-axis represents the threshold probability, corresponding to the risk level at which a patient would be referred, and the y-axis shows the net benefit, reflecting the balance between true positives and false positives. The model curve is compared with two reference strategies: treating all patients and treating none. A higher net benefit indicates greater clinical usefulness for decision-making

However, additional evaluation using the Brier score, calibration analysis, and decision curve analysis identified several areas requiring further improvement. The model demonstrated acceptable overall predictive performance, as reflected by a Brier score of 0.148. As shown in Fig. 1, calibration analysis further showed a systematic underestimation of risk (calibration intercept = 0.380), which may be considered conservative in the context of a screening model where missing true cases has significant clinical consequences. However, the calibration slope of 1.74 indicates that predicted probabilities were insufficiently dispersed, suggesting limited risk stratification across individuals. These findings imply that, while the model provides reasonably accurate overall risk estimates, further refinement may be needed to improve the distribution of predicted probabilities. In Fig. 2, decision curve analysis showed that the model provided a net benefit comparable to the “treat-all” strategy for threshold probabilities between 0% and 8%. This finding suggests that, within this range, the model does not clearly improve decision-making compared with treating all patients. Given that this low-threshold range reflects a clinical context in which minimizing missed cases is prioritized, the results highlight a need for further improvement in model performance to achieve a meaningful reduction in missed cases while maintaining acceptable levels of unnecessary interventions.

When comparing the NB, Linear SVM, and RF-all models, discriminative performance differed across the three approaches as reflected by ROC AUC. The RF-all model achieved the highest ROC AUC (84.7%), followed by the Linear SVM model (83.0%) and the NB model (79.1%). In contrast, sensitivity and specificity were generally comparable across models, with only modest differences observed. These results suggest that while all three approaches demonstrated similar threshold-dependent performance, the RF-all model provided superior overall discrimination. This pattern may reflect the advantage of nonlinear modeling approaches, suggesting that complex interactions among clinical features contribute to differentiating RA from non-RA conditions.

One of the key features of this study is the incorporation of physical findings into the predictive model. When comparing the Random Forest model using all predictors (RF-all) with models excluding physical findings (RF-noPhys) or laboratory variables (RF-noLab), the RF-all model demonstrated superior overall discriminative performance, with the highest ROC AUC (84.7%), compared with RF-noLab (82.8%) and RF-noPhys (80.1%). Although sensitivity and specificity were broadly comparable across models, the RF-noPhys model showed a more noticeable decline in both sensitivity and overall performance relative to RF-all. These findings suggest that the inclusion of physical findings contributes meaningfully to model performance, particularly in improving overall discrimination between RA and non-RA conditions, while supporting balanced classification performance. In a supplementary analysis, specific physical findings for non-RA, such as erythema, hypesthesia, and peripheral edema (Table 5), were identified, and including this information is considered to have contributed to the reduction of false positives.

However, extracting physical findings information was challenging. When extracting physical findings information as structured data from electronic medical records, some of the physical findings may have been overlooked. The existing physical findings extraction library could not extract the location of the physical findings. Therefore, physical findings that are not appropriate without location information could not be utilized as explanatory variables. For example, the sicca symptom of the mouth, which is specific to SjS, could not be extracted, leading to a higher number of false negatives in SjS cases (Table 6).

When differences in Random Forest modeling approaches were examined by comparing the RF-all and RF-stacked models, overall performance was comparable between the two models, as reflected by similar ROC AUC values (84.7% vs. 85.0%). Although the RF-stacked model showed higher sensitivity, this was accompanied by lower specificity, indicating differing classification characteristics rather than a clear overall advantage. However, the RF-stacked model was constructed using a two-stage approach, which enables intermediate classification into disease subtypes and may provide additional clinical insight. Inspection of the confusion matrix for the first-stage eight-disease classification revealed suboptimal classification performance for MCTD and SjS. This may be attributable to limitations in feature extraction, such as the inability to adequately capture sicca-related symptoms, which are important for distinguishing these conditions. In addition, as shown in Table 7, evaluation of the false-negative rates of the RF-all model demonstrated that PMR and SjS had particularly high false-negative rates. Differentiation between RA and PMR may be improved by incorporating features such as shoulder pain; however, this limitation may stem from the inability to capture the anatomical location of symptoms during the extraction of physical findings. In the present analysis, no substantial differences were observed between the RF-all and RF-stacked models. Nevertheless, further improvements in performance may be achievable through refinement of symptom extraction methods and feature representation.

With respect to differences in preprocessing of physical findings, the RF-binPhys model, in which missing values were imputed using the mode, showed comparable overall performance to the RF-all model. However, slight differences in classification performance and calibration were observed. These findings suggest that explicitly incorporating missingness information through a “not recorded” category may help preserve clinically meaningful information and improve model performance.

The observed differences in missingness patterns may reflect underlying clinical decision-making, whereby the selection of laboratory tests depends on the physician’s differential diagnosis. Variables such as reticulated platelet count, fibrinogen, serum calcium, serum phosphate, and urine protein, which are commonly used to evaluate multisystem involvement, showed higher observation rates in the non-RA group. This suggests that broader diagnostic workups were more frequently performed when non-RA conditions were suspected. As a result, directly incorporating missingness indicators could introduce bias by implicitly encoding clinicians’ diagnostic reasoning into the model. In the present study, however, missing values were imputed using the mean, which likely attenuated feature variability rather than amplifying such bias. This approach may have reduced the discriminative signal associated with certain variables, particularly in the RA group where missingness was more frequent. Therefore, although non-random missingness remains a potential limitation, its impact may be mitigated and is more likely to have led to conservative performance estimates rather than artificial inflation of model accuracy.

RA is one of the SARDs that has been extensively studied, particularly in terms of biomarkers and diagnostic models [13]. Although autoantibody testing is clinically useful, the accuracy of a single test is inherently limited. RA exhibits substantial molecular heterogeneity, and it has been suggested that biomarkers do not appear uniformly across patients, resulting in reduced sensitivity [13]. In contrast, machine learning–based approaches can incorporate many predictor variables and are expected to enable the construction of complex decision logic that accounts for disease heterogeneity. In this study, which focused on identifying non-RA among suspected SARDs, relatively high performance was achieved without using autoantibody tests.

This study has several limitations. First, the study cohort was restricted to patients with confirmed SARDs evaluated in a secondary care setting. As a result, the dataset did not include the broader primary care population presenting with musculoskeletal, inflammatory, infectious, degenerative, or other non‑autoimmune conditions. Therefore, the findings should be interpreted as reflecting internal predictive performance within a defined secondary care population. Second, external validation using an independent cohort was not performed, and the generalizability of the model therefore remains unassessed. Third, this was a single center retrospective study, which may limit representativeness and introduce selection bias. In addition, information on prior treatments, including corticosteroids, disease‑modifying antirheumatic drugs, and biologic agents, was unavailable for some patients and may have influenced clinical presentation and model performance. Future studies should focus on external validation in independent cohorts, prospective evaluation, and the development of models capable of handling a broader range of disease presentations.

Although the present analysis was confined to a secondary care setting with confirmed SARD diagnoses and evaluated internal predictive performance, the findings suggest potential directions for future research. With further advances in the extraction of physical findings from clinical records and validation using larger and more diverse datasets, similar screening models could be explored in broader clinical contexts, including primary care settings, in future studies.

## Supplementary Information

Below is the link to the electronic supplementary material.

Supplementary Material 1

## Data Availability

The datasets generated and analyzed during the current study are not publicly available due to participants of this study did not agree for their data to be shared publicly.

## References

[1] Goldblatt F, O'Neill SG. Clinical aspects of autoimmune rheumatic diseases. Lancet. 2013;382:797-808. 10.1016/S0140-6736(13)61499-3 23993190

[2] Oku K, Atsumi T. Rheumatology practice in Japan: challenges and opportunities. Rheumatol Int. 2019;39:1499-1505. 10.1007/s00296-019-04281-0 30888472

[3] Nakajima A, Sakai R, Inoue E, Harigai M. Geographic variations in rheumatoid arthritis treatment in Japan: a nationwide retrospective study using the national database of health insurance claims and specific health checkups of Japan. Mod Rheumatol. 2021;32:105-113. 10.1080/14397595.2021.1910615 33818268

[4] Suresh S, Charlton D, Snell EK, Laffoon M, Medsger TA, Zhu L, Domsic RT. Development of pulmonary hypertension in over one-third of patients with Th/To antibody–positive scleroderma in long-term follow-up. Arthritis Rheumatol. 2022;74:1580-1587. 10.1002/art.42152 PMC9477491 35467794

[5] Hassan AB, Hozayen RF, Mustafa ZS, Lundberg IE, Jahrami HA. The prevalence of pulmonary arterial hypertension in patients with mixed connective tissue disease: a systematic review and meta-analysis. Clin Exp Rheumatol. 2023. 10.55563/clinexprheumatol/srma43 37497727

[6] Ruiz-Irastorza G, Garmendia M, Villar I, Egurbide M-V, Aguirre C. Pulmonary hypertension in systemic lupus erythematosus: prevalence, predictors and diagnostic strategy. Autoimmun Rev. 2013;12:410-415. 10.1016/j.autrev.2012.07.010 22841984

[7] Jerjen R, Nikpour M, Krieg T, Denton CP, Saracino AM. Systemic sclerosis in adults. Part II: Management and therapeutics. J Am Acad Dermatol. 2022;87:957-978. 10.1016/j.jaad.2021.10.066 35131401

[8] Aletaha D, Neogi T, Silman AJ, Funovits J, Felson DT, Bingham CO, Birnbaum NS, Burmester GR, Bykerk VP, Cohen MD, Combe B, Costenbader KH, Dougados M, Emery P, Ferraccioli G, Hazes JM, Hobbs K, Huizinga TW, Kavanaugh A, Kay J, Kvien TK, Laing T, Mease P, Menard HA, Moreland LW, Naden RL, Pincus T, Smolen JS, Stanislawska-Biernat E, Symmons D, Tak PP, Upchurch KS, Vencovsky J, Wolfe F, Hawker G. 2010 rheumatoid arthritis classification criteria: an American College of Rheumatology/European League Against Rheumatism collaborative initiative. Ann Rheum Dis. 2010;69:1580-1588. 10.1136/ard.2010.138461 20699241

[9] Forrest IS, Petrazzini BO, Duffy Á, Park JK, O’Neal AJ, Jordan DM, Rocheleau G, Nadkarni GN, Cho JH, Blazer AD, Do R. A machine learning model identifies patients in need of autoimmune disease testing using electronic health records. Nat Commun. 2023;14:2385. 10.1038/s41467-023-37996-7 PMC10130143 37169741

[10] Junichi Kaburaki MK. How to Detect Early Stage Rheumatoid Arthritis (RA) in Persons Undergoing Ningen Dock (Health Evaluation and Promotion). Ningen Dock Int. 2014;1:24–33.

[11] Usui M, Aramaki E, Iwao T, Wakamiya S, Sakamoto T, Mochizuki M. Extraction and standardization of patient complaints from electronic medication histories for pharmacovigilance: natural language processing analysis in Japanese. JMIR Med Inform. 2018;6:e11021. 10.2196/11021 PMC6231790 30262450

[12] Kuhn M, Johnson K. Applied Predictive Modeling. Springer New York; 2013.

[13] Momtazmanesh S, Nowroozi A, Rezaei N. Artificial intelligence in rheumatoid arthritis: current status and future perspectives: a state-of-the-art review. Rheumatol Ther. 2022;9:1249-1304. 10.1007/s40744-022-00475-4 PMC9510088 35849321

